# Lung cancer and chronic obstructive pulmonary disease: From a clinical perspective

**DOI:** 10.18632/oncotarget.14505

**Published:** 2017-01-04

**Authors:** Jie Dai, Ping Yang, Angela Cox, Gening Jiang

**Affiliations:** ^1^ Department of Thoracic Surgery, Shanghai Pulmonary Hospital, Tongji University School of Medicine, Shanghai, China; ^2^ Department of Health Sciences Research, Division of Epidemiology, Mayo Clinic, Minnesota, United States of America; ^3^ Department of Oncology, University of Sheffield Medical School, Sheffield, United Kingdom

**Keywords:** chronic obstructive pulmonary disease, lung cancer, association

## Abstract

Chronic obstructive pulmonary disease (COPD) and lung cancer are devastating pulmonary diseases that commonly coexist and present a number of clinical challenges. COPD confers a higher risk for lung cancer development, but available chemopreventive measures remain rudimentary. Current studies have shown a marked benefit of cancer screening in the COPD population, although challenges remain, including the common underdiagnosis of COPD. COPD-associated lung cancer presents distinct clinical features. Treatment for lung cancer coexisting with COPD is challenging as COPD may increase postoperative morbidities and decrease survival. In this review, we outline current progress in the understanding of the clinical association between COPD and lung cancer, and suggest possible cancer prevention strategies in this patient population.

Chronic obstructive pulmonary disease (COPD) and lung cancer are both devastating pulmonary diseases [1], and they are projected to rank fourth and sixth cause of death in the next decades, respectively [2]. COPD is generally defined as chronic minimally reversible airflow obstruction on the basis of spirometry (post-bronchodilator forced expiratory volume in 1 second [FEV_1_]/forced vital capacity [FVC] less than 70%) [3], but it has now been recognized as a heterogeneous group of diseases, encompassing two well-characterized phenotypes: chronic bronchitis and emphysema [3]. Although many previous studies have investigated the role of COPD in the development and prognosis of lung cancer, their conflicting results have not been clearly understood, and some burgeoning areas of research, such as the incorporation of COPD into lung cancer screening criteria, still remain as a forum of open discussion.

This review will focus on the clinical epidemiologic association between COPD and lung cancer, ranging from lung cancer development and screening strategy to its treatment and prognosis in the setting of COPD, as well as cancer prevention strategies for this patient population (Figure 1).

**Figure 1:**
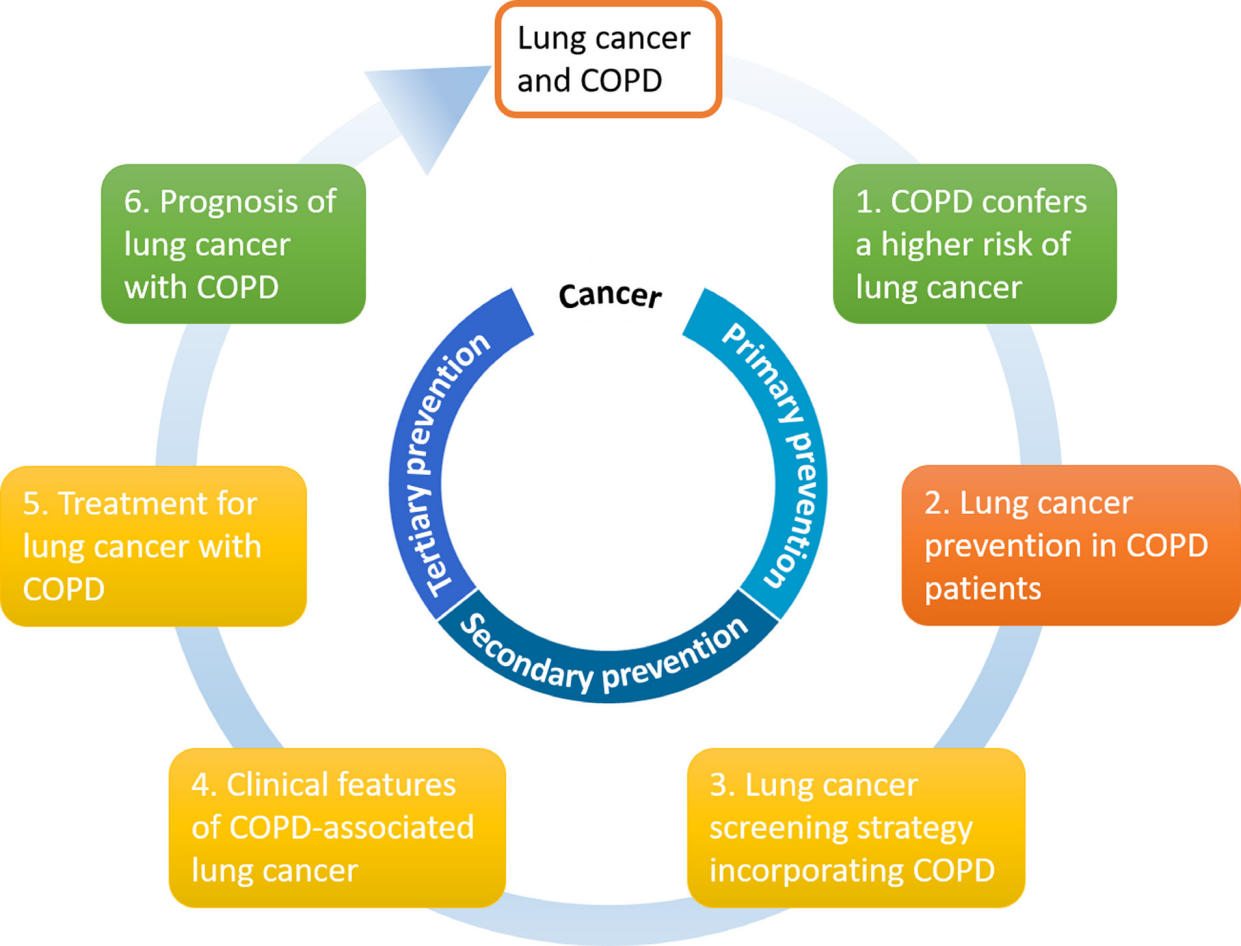
Clinical epidemiologic association between COPD and lung cancer in six areas The color codes refer to the current evidence showing the magnitude of association, where green indicates the association is clearly defined, amber is a debatable issue, and red is poorly understood. COPD: chronic obstructive pulmonary disease.

## COPD CONFERS A HIGHER RISK OF LUNG CANCER

Evidence on the association between COPD and lung cancer development has been extensively observed in population-based studies [4, 5], lung cancer screening trials [6–8], and case-control studies [9–12]. However, with the widespread use of computed tomography (CT), the research on the etiologic association has been gradually changing from spirometry-defined COPD to CT-diagnosed emphysema [13]. This has led to controversy about whether airflow obstruction on spirometry, or emphysema on CT scan, is the more important manifestation of COPD linked to an increased risk of lung cancer. Several studies [4–6, 9, 10, 14–16] have investigated the interaction between airflow obstruction and emphysema relative to lung cancer risk, but their results are still contradictory (Table 1). Reasons for these disparities may be threefold.

**Table 1:** Lung cancer risk according to airflow limitation and emphysema

Study	case *vs*. control	Sex, female	smoking status	Measurement of emphysema and associated lung cancer risk, OR (95%CI)	Measurement of airflow limitation and associated lung cancer risk, OR (95%CI)
Schwartz et al. [14] (2016, *n* = 1093)	341 *vs*. 752	54.3%	never: 2.8% ever: 97.2%	emphysema on qCT_-950HU_: 2.66 (1.80, 3.95)^¶^ emphysema by radiologist read: 1.80 (1.35, 2.41)^¶^ self-reported emphysema: 1.87 (1.25, 2.79)^¶^	spirometry FEV_1_/FVC<0.7: 1.98 (1.50, 2.61)^¶^ self-reported COPD: 1.43 (1.05, 1.94)^¶^
Wang et al. [15] (2012, *n* = 2201)	1069 *vs*. 1132	31.7%	never: 47.7% ever: 52.3%	self-reported emphysema: 1.92 (1.31, 2.81)^§^ self-reported emphysema: 1.55 (1.03, 2.32)^¶^	spirometry FEV_1_/FVC<0.7: 1.54 (1.21, 1.96)^§^ spirometry FEV_1_/FVC<0.7: 1.29 (1.00, 1.68)^¶^
Maldanado et al. [9] (2010, *n* = 441)	64 *vs*. 377	61.6%	current: 58.0% former: 42.0%	percent emphysema volume on qCT_-900HU_: 1.04 (0.82, 1.33)^¶^	spirometry FEV_1_/FVC continuous: 1.29 (1.02, 1.62)^¶^ spirometry FEV_1_% continuous: 1.15 (1.00, 1.32)^¶^
Schwartz et al. [5] (2009, *n* = 1126)	562 *vs*. 564	100%	never: 49.2% ex-smoker: 32.2% current: 38.6%	self-reported emphysema: 3.21 (1.60, 6.45)^¶^	self-reported COPD^#^: 1.67 (1.15, 2.41)^¶^
Koshiol et al. [4] (2009, *n* = 4042)	1934 *vs*. 2108	22.4%	never: 20.1% former: 43.0% current: 36.9%	self-reported emphysema: 3.8 (2.8, 5.1)^¶^ self-reported emphysema: 1.9 (1.4, 2.7)^‡^	self-reported COPD^#^: 4.1 (3.4, 4.9)^¶^ self-reported COPD^#^: 2.5 (2.0, 3.1)^‡^
Wilson et al. [6] (2008, *n* = 3638)	99 *vs*. 3539	48.6%	current: 60.2% ex-smoker: 39.8%	emphysema by radiologist read: 4.39 (2.76, 6.99)^§^ emphysema by radiologist read: 3.56 (2.21, 5.73)^¶^ emphysema by radiologist read: 3.14 (1.91, 5.15)^‡^	spirometry FEV_1_/FVC<0.7: 2.89 (1.89, 4.43)^§^ spirometry FEV_1_/FVC<0.7: 2.09 (1.33, 3.27)^¶^ spirometry FEV_1_/FVC<0.7: 1.41 (0.87, 2.29)^‡^
de Torres et al. [10] (2007, *n* = 1166)	23 *vs*. 1143	26%	former: 100%	emphysema by radiologist read: 3.33 (1.41, 7.85)^§^ emphysema by radiologist read: 3.13 (1.32, 7.44)^¶^ emphysema by radiologist read: 2.51 (1.01, 6.23)‡	spirometry FEV_1_/FVC<0.7: 4.83 (2.05, 11.41)^§^ spirometry FEV_1_/FVC<0.7: 2.89 (1.14, 7.27)^¶^ spirometry FEV_1_/FVC<0.7: 2.10 (0.79, 5.58)^‡^
Kishi et al. [16] (2002, *n* = 120)	24 *vs*. 96	58.3%	former: 45% current: 55%	percent emphysema volume on qCT_-900HU_: 1.1 (0.6, 1.9)^¶^	spirometry FEV_1_/FVC continuous: 1.4 (1.0, 2.2)^¶^ spirometry FEV_1_% continuous: 1.2 (1.0, 1.5)^¶^

OR: odds ratio; CI: confidence interval; FEV_1_: post-bronchodilator forced expiratory volume in 1 second; FVC: forced vital capacity; COPD: chronic obstructive pulmonary disease.

^§^unadjusted analysis; ^¶^adjusted for patient demographics; ^‡^adjusted for patient demographics, and emphysema or airflow limitation, as appropriate.

^#^ including reports of emphysema, COPD, and/or chronic bronchitis.

Firstly, patient demographics may have residual effects even after adjustment. Several studies revealed that the magnitude of the COPD lung cancer association is influenced by patient's gender [5, 17], smoking habit [4, 7, 11] and other respiratory disease [18], where the strength of association increases in women, never-smokers and the co-existence with pneumonia. Secondly, the method to determine emphysema seems to be important. Automated analysis virtually eliminates subjectivity in the estimation of emphysema [9, 19], whereas visual assessment detects clinically meaningful emphysema and avoids incorrect interpretation by computer software [20, 21]. A recent meta-analysis showed that the association with lung cancer was only significant for visually-determined emphysema [22]. Thirdly, variable definitions of airflow obstruction can give rise to conflicting results. A ratio of FEV_1_ and FVC of less than 0.7 is generally used to define airflow obstruction [10]; however, other indices, such as FEV_1_/FVC under the lower limit of normal criteria, and reduction of FEV_1_% predicted, have also been considered indicative of airway obstruction [17, 23, 24]. In addition to these three main factors, timing of COPD diagnosis [4, 11], degree of airflow obstruction [9, 25] and severity of emphysema [6, 20] have also been reported to exert a remarkable effect on the significance of the impact of COPD and/or emphysema on lung cancer risk. Although no solid evidence is available at present to clearly distinguish the roles of airflow obstruction and emphysema in lung cancer development, it is certain that the highest lung cancer risk occurs when airflow obstruction and emphysema coexist [10, 14]. Therefore, both airflow obstruction and emphysema should be regarded as risk factors for lung cancer, and as such, could help identify individuals who may need active interventions to preempt tumorigenesis and target population who may benefit most from cancer screening.

## LUNG CANCER PREVENTION IN COPD PATIENTS

Lung cancer is one of the most common causes of death among COPD patients [26], and continued cigarette smoking poses an additional lung cancer risk in patients with preexisting COPD [27]. So the first priority of lung cancer preventive measures is smoking cessation. A population-based cohort study with 31-year follow-up demonstrated that participants who quit smoking reduced their lung cancer risk by 50% [28]. The Lung Health Study, which enrolled 5, 887 smokers with asymptomatic airflow obstruction (FEV_1_/FVC ≤70%), further confirmed that lung cancer mortality could be improved most by smoking cessation [29].

Secondhand tobacco smoke (SHS) exposure is another important risk factor for lung cancer [30]. Henschke and colleagues revealed that emphysema increased the risk of lung cancer in never smokers while the SHS exposure was an independent indicator of emphysema [7]. In addition, Kim and colleagues pooled data from 18 case-control studies in the International Lung Cancer Consortium [30], and found that, among both ever smokers and never smokers, risk of lung cancer increased with increasing years of SHS exposure. Therefore, SHS exposure should be avoided in COPD patients at the same time.

Inhaled corticosteroids (ICS), which are commonly prescribed to COPD patients, are now showing a potential cancer prevention effect [31]. A nested case-control study of patients with COPD demonstrated that regular use of ICS was significantly associated with a decreased lung cancer risk [32]. Moreover, Parimon and colleagues observed a dose-response relationship where higher doses of ICS (≥1, 200 ug/d) conferred a risk reduction of lung cancer of 61% in COPD patients [33]. Statins could attenuate the inflammation in COPD [34, 35] and have potential anticancer effects [36, 37]; however, two recent meta-analyses indicated no significant association between statin use and the risk of lung cancer [38, 39]. For patients with COPD who are already at an increased risk of developing lung cancer, Liu and colleagues found that COPD patients who used statins exhibited a 63% reduced lung cancer risk [40]. Although the data on chemopreventive agents (i.e., ICS and statin) at present are not as definitive as smoking cessation, it is axiomatic that the above-mentioned measures could help not only reduce the incidence of lung cancer but also mitigate the progression of COPD.

## LUNG CANCER SCREENING STRATEGY INCORPORATING COPD

Low-dose CT (LDCT) is recommended for lung cancer screening by the United States Preventive Services Task Force (USPSTF) [41]; however, a recent report showed an increasing number of patients with newly diagnosed lung cancer falling outside the population suggested by USPSTF eligibility criteria [42]. Since patients with COPD, regardless of airflow obstruction or emphysema, are at higher risk for developing lung cancer [8, 12], several studies have targeted this population as a candidate for lung cancer screening [8, 23, 43]. Lowry and colleagues compared the health benefits of different screening programs, and the results showed that a program using lower pack-year thresholds (≥1 pack-year) for individuals with COPD could yield higher life expectancy gains than USPSTF using smoking history alone [23]. Meanwhile, the detection rate and diagnostic precision could be improved by adding CT-detected emphysema as a complementary entry criterion to the National Lung Screening Trial (NLST) [8]. With respect to survival advantage, results from the Danish Lung Cancer Screening Trial indicated a favorable effect of screening on lung cancer mortality in COPD patients [44]. In order to increase the implementation of lung cancer screening among COPD patients, however, several aspects should be considered.

(i) *Underdiagnosis and misdiagnosis of COPD*. It has been demonstrated that COPD is remarkably under-diagnosed worldwide, with an estimated rate of underdiagnosis of 71.2%-81.4% [45, 46]. Misdiagnosis of COPD also poses a clinical challenge; 30.4%-40% of patients with a prior COPD diagnosis were found to have normal lung function on spirometry [47, 48]. As a result, recommendation for lung cancer screening in self-reported COPD would only benefit a limited population, leaving four-fifths of cases unrecognized and one-third over-treated. Evidence on airflow obstruction and/or emphysema are therefore the ideal surrogates in the context of lung cancer screening. Young and colleagues [49] argued for a widespread use of spirometry screening for airflow obstruction in asymptomatic smokers, in an attempt to appropriately evaluate the prevalence of COPD and detect individuals at an increased risk for lung cancer early. Some identified determinants of under-diagnosed COPD include male sex, lower level of education, being of ethnic minority, and lower comorbidity burden [45, 46, 50], while younger age, being overweight, and higher levels of comorbidities are risk factors for COPD misdiagnosis [50–52]. Therefore, individuals with these characteristics should be offered spirometry for a correct diagnosis of COPD, which in turn will allow for further risk stratification in lung cancer screening [53].

(ii) *Further risk assessment*. As COPD is identified as a driving factor in lung cancer, a refined risk stratification among patients with preexisting COPD can further improve the cost-effectiveness of CT screening and avoid unnecessary radiation exposure [54]. De-Torres and colleagues explored risk factors associated with lung cancer development in a cohort of outpatients with COPD, and identified four independent predictors: baseline age, body mass index (BMI), predicted percentage of diffusion capacity for carbon monoxide (DLCO%), and GOLD stages [25]. Subsequently, the COPD-specific score (COPD-LUCSS) was developed to predict lung cancer risk for patients with COPD [27]. COPD-LUCSS is determined by four parameters: age >60, BMI <25kg/m^2^, pack-years >60, and presence of radiological emphysema, with a total range from 0 to 10 points [27]. In comparison with low-risk patients (scores 0-6), lung cancer risk increased 3.5-fold in the high-risk category (scores 7-10). Therefore, COPD patients who are considered at low risk of lung cancer may need less frequent screening compared to those at high risk, resulting in further reductions in cost and screening-related harms.

(iii) *Over-diagnosis of pulmonary nodules*. Over-diagnosis refers to excess lung cancer detected by screening that would not affect the patient during their lifetime if left untreated [55], which may incur additional cost, patient anxiety, and potential morbidities related to subsequent diagnostic procedures [56]. It is estimated that over-diagnosis accounted for as much as 18.5% of all lung cancers detected by LDCT [57]. De-Torres and colleagues found that screening in COPD patients resulted in a higher detection rate of early-stage lung cancer, without showing a significant “histology shift” towards over-diagnosis [43, 58]. Young and colleagues examined the effect of COPD on over-diagnosis and demonstrated that LDCT screening in COPD patients yielded a doubling of lung cancer incidence without apparent over-diagnosis, whereas in non-COPD patients, the stage shift was counterbalanced by the excess diagnosis of bronchioloalveolar carcinoma [59]. The available data suggest that lung cancer screening in individuals with COPD may contribute to a high rate of diagnosis of lung cancer at curable stage while minimizing over-diagnosis [59, 60].

(iv) *Competing causes of mortality/morbidity*. The USPSTF recommended that screening should not be offered to people who have substantial comorbid conditions with limited life expectancy [41]. Sin and colleagues reviewed the underlying causes of death in COPD patients and reported that the main cause of death for mild-to-moderate COPD was lung cancer, while for more advanced COPD, respiratory failure was the predominant cause [26]. As regards benefit from screening, De-Torres and colleagues explored the impact of screening on lung cancer mortality in patients with mild-to-moderate COPD [43], and the results showed that the mortality incidence densities from lung cancer were significantly lower in the screening group than in the control group (unscreened COPD), justifying active screening in patients with milder COPD. However, screening patients with more severe COPD may reduce cost-effectiveness because the benefits could be surpassed by other competing causes of death inherent to COPD [61]. In preliminary data from a post-hoc analysis of the NLST, it has been shown that the lung cancer specific mortality reduction in screening participants with COPD was approximately one half that of those without COPD (15% vs 28% respectively), suggesting the benefits of CT screening in COPD may be diluted by competing causes of death [62, 63]. Therefore, the trade-off between the potential benefits and harms should be considered by participants and their health providers together, when considering lung cancer screening in patients with more severe COPD [54].

## DIFFERENT CLINICAL AND MOLECULAR FEATURES OF COPD-ASSOCIATED LUNG CANCER

Squamous cell carcinoma is more commonly seen in the setting of COPD and/or emphysema [25, 64], and lung cancer arising in COPD is more likely to be centrally located [65]. However, lower emphysema grade tends to be associated with central location of lung cancer, while higher grade with peripheral location [65]. When the extent of emphysema was quantified regionally, a strong association was found for cancer being located in the area with the highest degree of emphysema [66].

In histopathologic analysis, Schiavon and colleagues described that COPD-associated adenocarcinoma tended to manifest less invasive characteristics, such as increased lepidic component and lower cell proliferation, as compared to COPD-free adenocarcinoma [67]. However, Murakami and colleagues commented that cancer arising in emphysema possessed a more aggressive nature [68], because the matrix metalloproteinase, which was widely up-regulated in emphysematous lungs, was associated with the occurrence of lymphovascular invasion and postoperative recurrence [68, 69]. Moreover, in post-hoc analyses of two CT screening studies, it has been shown that smokers with impaired lung function had shorter volume doubling times of pulmonary nodules (more aggressiveness) and less prevalence of indolent lung cancers, suggesting COPD is a useful clinical marker of aggressive lung cancer [70–72]. With regard to molecular features, several studies found that both EGFR mutations and ALK rearrangements were less prevalent in COPD-associated lung cancer [73, 74], and the presence of EGFR mutations was inversely correlated with the severity of airflow limitation [73]. In contrast, KRAS mutations were independent of COPD status [75, 76]. It is worth mentioning, however, that the traits of driver genes alternations in lung cancer with COPD could be partly due to their associations with patient clinical characteristics [75].

## TREATMENT FOR LUNG CANCER WITH COPD

Major lung resection is the best option for cure in lung cancer patients; however, it has been reported that about one-third of patients with comorbid COPD may be ineligible for surgery for lung cancer that would otherwise be technically operable, due to poor physical condition [74]. Furthermore, the frequencies of all postoperative pulmonary complications (PPCs), including pneumonia (10.1%-16.2% in COPD patients following lung cancer surgery) [77–79], atelectasis (3.5%-15.4%) [79, 80], empyema (2.2%-8.3%) [78], and persistent air leak (12%-16.2%) [77, 81], were often higher in COPD patients. Therefore, an accurate risk assessment in patients with lung cancer coexisting with COPD is critically important, in order to optimize treatment for these patients.

(i) *Identifying risk factors for PPCs*. There are only a small number of studies to date that have investigated risk factors for PPCs in COPD patients undergoing lung cancer surgery. Kim and colleagues, in their prospective study, reported that the incidence of PPCs was higher in patients with COPD but not different between COPD grades (FEV_1_% ≥70% vs. FEV_1_% <70%) or symptom burden (less symptoms vs. more symptoms) [79]. Multivariate analysis revealed that lower BMI, reduced DLCO%, and operation time were significant predictors of PPCs in COPD patients receiving cancer resection. In cardiopulmonary exercise testing, Rodrigues and colleagues found that the cutoff value of 61% for peak oxygen uptake was a significant discriminator between COPD patients with and without complications following tumor resection [82]. With regard to surgical approach, Jeon and colleagues performed a propensity score-matched analysis and demonstrated that video-assisted thoracoscopic surgery in lung cancer patients with comorbid COPD could reduce PPCs compared with thoracotomy [83].

(ii) *Effective perioperative management*. Medical management for COPD, smoking abstinence, and pulmonary rehabilitation are three major effective strategies to improve postoperative outcomes [84, 85]. Pharmacologic therapy for COPD, such as bronchodilators and ICS, can help reduce symptoms, prevent exacerbations, and thus increase perioperative safety [79, 86]. The use of ICS has been justified by a recent study that revealed no relationship between the perioperative ICS administration and the incidences of PPCs in COPD patients receiving pulmonary resection for lung cancer [87].

It is clear that smoking cessation should be advocated preoperatively. However, the timing of tobacco cessation is still controversial. Although a general trend was observed for decreasing PPCs with an increase in the length of cessation prior to surgery [88], some studies, were not as supportive, showing a higher risk for PPCs in patients who had quit smoking in the immediate preoperative period [89, 90]. Hypothetical explanations for this increased risk may relate to the effect of nicotine withdrawal and increased sputum volume caused by the reduction in irritant-induced coughing, before the recovery of ciliary function [91]. Therefore, smoking cessation should be encouraged with sufficient duration (2-4 weeks) before surgery [85].

The effect of preoperative pulmonary rehabilitation has now been demonstrated in patients with COPD undergoing lung cancer resection [92–94]. Divisi and colleagues targeted 27 patients with compromised lung function and observed a significant increase in FEV_1_ (from mean FEV_1_ of 1.14L to 1.65L) after a 4-week preoperative pulmonary rehabilitation [94]. Moreover, pulmonary rehabilitation is shown to decrease postoperative complications as well as length of hospital stay [95]. Despite the small sample size included in previous studies [94–96], the documented benefits underscore the importance of pulmonary rehabilitation for patients with advanced COPD prior to lung cancer surgery, to help to reduce the function limitations of inoperability.

(iii) *Predictors of lung volume reduction effect*. Patients with lung cancer and COPD receiving cancer resection may have a minimal loss, or improvement, in postoperative pulmonary function, which is referred to as the “lung volume reduction effect” [97, 98]. Various methods have been reported to determine potential candidates who are more likely to have the functional benefit. Korst and colleagues defined the COPD index, a scoring system combining preoperative FEV_1_% predicted and FEV_1_/FVC [99], and found that COPD index <1.0 was a good indicator of an improvement of pulmonary function following lobectomy. Sekine and colleagues documented a greater actual postoperative FEV_1_ than predicted in COPD patients with lobectomy of lower portion [100]. Furthermore, quantitative analysis of radiologic emphysema could characterize the respiratory dynamics underlying the volume reduction effect [101].

(iv) *Non-surgical treatment*. Patients who are unfit for surgery due to poor lung function and/or COPD-related systemic comorbidities (such as ischemic cardiac disease) could benefit from stereotactic body radiotherapy (SBRT), which has been shown as a safe and effective alternative treatment for early-stage lung cancer [102]. A recent study by Pamla and colleagues reported a 3-year actual local control rate of 89% in stage I non-small cell lung cancer (NSCLC) patients with concomitant COPD (GOLD class III/IV) after SBRT [103], and a subsequent systematic review demonstrated comparable outcomes between SBRT and surgery in this patient population [103]. The toxicity following SBRT was tolerable, and even milder in patients with COPD than those with normal lung function [104]. Data on the effectiveness of chemotherapy in COPD-associated lung cancer remain limited, although COPD has been reported to increase the risk of chemotherapy-induced febrile neutropenia [105].

(v) *Multidisciplinary treatment (MDT)*. MDT can improve adherence to evidence-based guidelines and timeliness of care for lung cancer patients [106]. In addition, in the setting of advanced NSCLC, MDT has been reportedly associated with a better survival rate [107]. Since lung cancer and COPD often coexist, pulmonologists could provide prompt diagnosis for lung cancer and effective management of pulmonary comorbidities [108]. Data from the Surveillance, Epidemiology and End Results (SEER) database showed that the involvement of pulmonologists in the care of patients with early-stage NSCLC and COPD could increase surgical resection rate and reduce mortality risk [109]. Thus, MDT should be incorporated into the treatment of lung cancer concomitant with COPD.

## PROGNOSIS OF LUNG CANCER WITH COPD

The prognostic significance of COPD in lung cancer remains equivocal. Most studies found that COPD exerted an unfavorable effect on lung cancer prognosis [78, 110, 111], while others did not [112, 113]. Two recent meta-analyses indicated COPD as an adverse prognostic predictor, but the results suffered from a high level of heterogeneity between studies [114, 115]. The heterogeneity of effect size is possibly subject to cancer stage, treatment modality and status of COPD *per se* [115]. Zhai and colleagues found that coexisting COPD was associated with worse survival in patients with early-stage NSCLC undergoing surgical resection [110]. However, this association was insignificant in the study by Izquierdo and colleagues [112], who targeted patients with advanced lung cancer (stage IIIB/IV) treated with chemotherapy. In terms of COPD grade, there was a more apparent decrease in survival for patients with severe COPD, but not for those with mild-to-moderate COPD, as compared to non-COPD patients following lung cancer resection [78, 116]. In addition, quantitative analysis of emphysema on CT demonstrated a direct association with lung cancer mortality [21].

Recently, a prognostic model (COPD-LUCSS-DLCO) was designed to identify patients with COPD at high risk of lung cancer death [117]. Patients were divided into two risk groups based on a composite score determined by patient's age (2.5 points), BMI (1.5 points), pack-year of smoking (1 point), and DLCO% (3 points), where the high-risk group (scores 3.5-8) conferred a 2.4-fold increased risk of death when compared to the low-risk group (scores 0-3). In addition, research from the linked SEER-Medicare Database demonstrated the addition of comorbid COPD to a comprehensive model could improve prognostication over similar models using cancer information alone [118].

With regard to health-related quality of life, Pompili and colleagues performed a propensity score-matched analysis among patients undergoing lobectomy for lung cancer, and found that patients with COPD experienced a comparable postoperative quality of life to matched patients without COPD [119]. Pompeo and colleagues studied patients who underwent tailored combined surgery for both stage I NSCLC and severe emphysema, and demonstrated a significant improvement in general health domain based on short-form 36 item questionnaire after surgery, associated with improvements in dyspnea index and exercise capacity [120].

## CANCER PREVENTION STRATEGY AND FUTURE EFFORTS

Lung cancer prevention strategies should be emphasized and encouraged throughout the entire disease process. Primary prevention is aimed at limiting the incidence of lung cancer. COPD, characterized as either airflow obstruction or emphysema, is an important predisposing factor for lung cancer development. Thus, a primary aim is to control the additional exposures (such as smoking and SHS exposure) which contribute to COPD, lung cancer, and the progression from COPD to lung cancer. The use of chemopreventive agents such as ICS and statin remain relatively rudimentary in COPD patients, and should be tested in prospective, controlled trials. Secondary prevention refers to the early detection of lung cancer at a pre-clinical phase, and lung cancer screening represents the most important component of this approach. The current available evidence shows that lung cancer screening in COPD patients confers a high detection rate of cancer at early stage (stage shift), and reduces lung cancer mortality. Nevertheless, some screening-related issues (e.g., underdiagnosis of COPD and potential benefit offset) ought to be recognized and discussed in the future. With respect to clinical features, lung cancer in COPD is quite distinct from that in non-COPD, highlighting the demand for a designated screening criteria as well as a tailored treatment algorithm in this patient population. Tertiary prevention points to the execution of treatment and rehabilitation with the principal aim of alleviating disability and improving the outcomes of illness. Surgery for lung cancer in COPD may have a lung volume reduction effect. Precise risk assessment, optimal preoperative management (smoking cessation, medical treatment for COPD, and pulmonary rehabilitation), and MDT care are critically important before surgery. Meanwhile, the recognition of the effect of COPD on lung cancer prognosis enables refined prognostication and thus allows for personalised clinical decision-making. Increasing understanding of the relationship between COPD and lung cancer will allow the development of better cancer preventive strategies and ultimately will improve the outcomes of this patient population.

## Abbreviations

COPD: chronic obstructive pulmonary disease
DLCO: diffusion capacity of lung for carbon monoxide
FEV_1_: forced expiratory volume in one second
FVC: forced vital capacity
GOLD: Global Initiative for Chronic Obstructive Lung Disease
ICS: inhaled corticosteroids
LDCT: low-dose computed tomography
MDT: multidisciplinary treatment
NSCLC: non-small cell lung cancer
PPC: postoperative pulmonary complication
SHS: secondhand tobacco smoke
USPSTF: United States Preventive Services Task Force
